# Expansion of the SOS regulon of *Vibrio cholerae* through extensive transcriptome analysis and experimental validation

**DOI:** 10.1186/s12864-018-4716-8

**Published:** 2018-05-21

**Authors:** Evelyne Krin, Sebastian Aguilar Pierlé, Odile Sismeiro, Bernd Jagla, Marie-Agnès Dillies, Hugo Varet, Oihane Irazoki, Susana Campoy, Zoé Rouy, Stéphane Cruveiller, Claudine Médigue, Jean-Yves Coppée, Didier Mazel

**Affiliations:** 1 Département Génomes et Génétique, Institut Pasteur, Unité de Plasticité du Génome Bactérien, Paris, France; 2 CNRS, UMR 3525, Paris, France; 3 Institut Pasteur, Transcriptome and EpiGenome, Biomics Center for Innovation and Technological Research, Paris, France; 4 Departament de Genètica i de Microbiologia, Universitat Autònoma de Barcelona, Cerdanyola del Vallès, Bellaterra, Spain; 5 UMR 8030, CNRS, CEA, Institut de Biologie François Jacob - Genoscope, Laboratoire d’Analyses Bioinformatiques pour la Génomique et le Métabolisme, Université Evry-Val-d’Essonne, Evry, France; 6 Present adress: Institut Pasteur, Biomarker Discovery Platform, UtechS CB and Hub Bioinformatique et Biostatistique – C3BI, USR 3756 IP CNRS, Paris, France; 7 Present adress: Institut Pasteur, Hub Bioinformatique et Biostatistique – C3BI, USR 3756 IP CNRS, Paris, France

**Keywords:** SOS response, DNA repair, Transcriptome, ncRNA, nrdA, nrdB

## Abstract

**Background:**

The SOS response is an almost ubiquitous response of cells to genotoxic stresses. The full complement of genes in the SOS regulon for *Vibrio* species has only been addressed through bioinformatic analyses predicting LexA binding box consensus and in vitro validation. Here, we perform whole transcriptome sequencing from *Vibrio cholerae* treated with mitomycin C as an SOS inducer to characterize the SOS regulon and other pathways affected by this treatment.

**Results:**

Comprehensive transcriptional profiling allowed us to define the full landscape of promoters and transcripts active in *V. cholerae*. We performed extensive transcription start site (TSS) mapping as well as detection/quantification of the coding and non-coding RNA (ncRNA) repertoire in strain N16961. To improve TSS detection, we developed a new technique to treat RNA extracted from cells grown in various conditions. This allowed for identification of 3078 TSSs with an average 5’UTR of 116 nucleotides, and peak distribution between 16 and 64 nucleotides; as well as 629 ncRNAs. Mitomycin C treatment induced transcription of 737 genes and 28 ncRNAs at least 2 fold, while it repressed 231 genes and 17 ncRNAs. Data analysis revealed that in addition to the core genes known to integrate the SOS regulon, several metabolic pathways were induced. This study allowed for expansion of the *Vibrio* SOS regulon, as twelve genes (*ubiEJB, tatABC, smpA, cep*, *VC0091*, *VC1190*, *VC1369–1370*) were found to be co-induced with their adjacent canonical SOS regulon gene(s), through transcriptional read-through. Characterization of UV and mitomycin C susceptibility for mutants of these newly identified SOS regulon genes and other highly induced genes and ncRNAs confirmed their role in DNA damage rescue and protection.

**Conclusions:**

We show that genotoxic stress induces a pervasive transcriptional response, affecting almost 20% of the *V. cholerae* genes. We also demonstrate that the SOS regulon is larger than previously known, and its syntenic organization is conserved among *Vibrio* species. Furthermore, this specific co-localization is found in other γ-proteobacteria for genes *recN-smpA* and *rmuC-tatABC*, suggesting SOS regulon conservation in this phylum. Finally, we comment on the limitations of widespread NGS approaches for identification of all RNA species in bacteria.

**Electronic supplementary material:**

The online version of this article (10.1186/s12864-018-4716-8) contains supplementary material, which is available to authorized users.

## Background

*Vibrio cholerae* is the bacterium responsible for cholera, causing severe diarrheal disease and dehydration in vulnerable populations. During the course of its life cycle, *V. cholerae* alternates between various environmental growth conditions: freshwater, brackish water, sea and, finally, transit through the “gastric acidity barrier” before colonizing the intestinal tract during human infection [1]. In these environments, *V. cholerae* is exposed to various stresses, which has led to selection of several mechanisms of adaptation that allow the bacterium to thrive in these strenuous conditions. Among these, the SOS response plays a central role. Indeed, it is induced when a high level of single-stranded DNA (ssDNA) is present in the cell, as a consequence of DNA damage or during horizontal transfer through conjugation and transformation [2–4]. This leads to RecA proteins forming nucleofilaments on ssDNA, which in turn catalyzes self-cleavage of the LexA repressor, releasing expression of the SOS regulon [5]. These genes are characterized by the presence of a LexA box near or overlapping their promoter [2]. Some genes in this regulon are ubiquitously conserved, such as *lexA, recA*, *dinB*, *uvrD* or *ruvAB*, while some are species specific. A bioinformatics analysis of LexA binding box distribution in *Vibrio* genomes revealed two such *Vibrio* specific gene candidates, *unfA* and *unfB* [5]. These genes exhibit a binding box recognized by LexA upstream of their start codon. However, no induction has been shown for them during SOS triggering and their role in this pathway is still unknown [5]. In addition, the collection of genes and non-coding RNAs (ncRNAs) upregulated during the SOS response has yet to be experimentally established in *V. cholerae*.

In recent years, the importance of the regulatory roles of ncRNAs in bacteria has become more and more prominent. Several ncRNAs are conserved among γ-proteobacteria, such as those from the *csrB* family (three copies in *V. cholerae* [6]). The latter block the translation inhibiting function of CsrA, involved in virulence and biofilm formation. Another example is *ryhB*, which participates in positive regulation of TCA cycle activity, resistance to oxidative stress and iron storage in many bacteria [7, 8]. Others are likely specific to all *Vibrio* species, such as *tfoR*, which acts on the natural competence activator TfoX [9]. Several ncRNAs act as positive regulators by binding to their mRNA targets. Indeed, some mRNAs have secondary structures that sequester the RBS, limiting their accessibility. Alternatively, ncRNA binding to the RNase E entry site can render unstable mRNAs more suitable for expression [10]. In contrast, many ncRNAs act on their target genes by pairing to their mRNA, leading to double-stranded RNA formation and subsequent mRNA degradation [11]. Three studies that explore the ncRNAs contents of *V. cholerae* have been published [12–14]. However, their results show a lot of incongruences in terms of ncRNA repertoire, and, as previously notified by Toffano-Nioche and collaborators [15], many candidates likely correspond to truncated forms of the same RNA.

In order to clarify these discrepancies, and also to precisely establish the extent of the SOS regulon, and of the SOS response, we performed a whole transcriptome analysis, including transcriptional start site (TSS) mapping. For this we used a new protocol that prevents degradation of the full RNA molecule, all along the RNA molecule and from the native 5’RNA extremities. This allowed us to define the start positions of the transcripts and the set of native ncRNAs at a genomic scale in the N16961 strain of *V. cholerae,* using the longest possible transcripts*.* We describe here a global study of the SOS response in *V. cholerae* after induction with subinhibitory concentrations of mitomycin C (MMC), which reveals the presence of twelve additional genes to the core SOS regulon issued from previous bioinformatics studies of this nearly ubiquitous response [5]. These genes are expressed through transcriptional readthrough from their neighboring SOS genes. Interestingly, syntenic conservation of the genetic neighborhood of four of these new SOS genes suggests that they are likely also part of the SOS response in γ-proteobacteria, beyond the *Vibrio* genus.

## Methods

### Bacterial strains and growth conditions

The *V. cholerae* N16961 strain was used in this study. Gene deletions were performed using 500-base pair homologous regions upstream and downstream of the gene of interest cloned into the pSW7848 plasmid. The plasmid was then introduced in the *V. cholerae* N16961 strain. After homologous recombination with the chromosome, the targeted gene was deleted as previously described [3, 16, 17]. Mutant strains are described in Additional file 1. Strains were grown at 37 °C in Marine Broth (Roth) (NaCl 19.4 g/l: 332 mM) until exponential growth phase, with or without addition of 200 ng/ml MMC for SOS induction, for 90 min, as previously described [17]; in Luria Broth (BD) with high NaCl concentration (NaCl 19.4 g/l: 332 mM) until exponential and stationary growth phases, and at stationary phase during anaerobic growth; in 1% Bacto-Tryptone (BD) with 5 g/l NaCl and 1% succinate until stationary growth phase; in M63 minimal medium (supplemented with 0.4% succinate, 100 μg/ml asparagine and 10 μg/ml vitamin B1) during exponential growth phase.

### RNA preparation

Total RNA was purified from frozen bacteria pellets through trizol extraction using 1 ml of the reagent during 5 min at room temperature. Membrane debris were removed with a 15 min centrifugation at 13000 g at 4 °C. All reagents were gently mixed and incubated for 5 min at room temperature before centrifugation. All subsequent centrifugations lasted 5 min. After centrifugation with 400 μl of chloroform/isoamyl alcohol (24/1), the aqueous phase was treated with 500 μl acidic phenol and an additional 300 μl chloroform/isoamyl alcohol mix. Total RNA was then washed a second time with 200 μl chloroform/isoamyl alcohol mix, and then precipitated with 250 μl isopropanol for 15 min on ice. Total RNA was later centrifuged 15 min at 13000 g at 4 °C; pellets were washed with 1 ml 70% ethanol and centrifuged 5 min at 13000 g at 4 °C. Pellets were then air dried and resuspended for 15 min at 60 °C in 200 μl Tris 10 mM EDTA 0.1 mM pH 7.4. Genomic DNA was removed using 2 μl Turbo DNase (AMBION) for 30 min at 37 °C. Total RNA was quantified using Thermo Fisher’s NanoDrop. RNA integrity was verified on a Nano6000 RNA chip using the Bioanalyzer 2100 (Agilent). mRNA enrichment was performed with the MICROBExpress Kit (Ambion) from 7 μg of total RNA per sample. Depletion of 16S and 23S ribosomal RNAs was confirmed on a Nano6000 RNA chip with the Bioanalyzer 2100 (Agilent).

### 5’end-RNA-seq library

Two different treatments were combined: ± Terminator 5’-Phosphate-Dependent Exonuclease (TEX) [18] and ± Tobacco Acid Pyrophosphatase (TAP) [19]. Depleted RNA samples, containing the equivalent of 7 μg of total RNA, were denatured and treated with 1 unit of TEX (Epicentre) during 90 min at 30 °C and/or 10 units of TAP (Epicentre), for 1 h at 37 °C. Sample purification was performed using a phenol-chloroform extraction followed by an ethanol precipitation after each enzymatic treatment. Samples without TEX or TAP treatments underwent all the incubation and purification steps in parallel with the treated ones. All the samples were ligated with an excess of the 5′ adapter, 5′- GUU CAG AGU UCU ACA GUC CGA CGA UC – 3′ (Illumina TruSeq Small RNA kit). Reverse transcription was performed at 50 °C for 1 h using Superscript III (Invitrogen) and a random primer (RPO primer: 5′CCTTGGCACCCGAGAATTCCANNNNNN-3′). First strand cDNA/RNA hybrids were then run on a 2% Low Range Agarose gel (Biorad). cDNAs ranging from 200 to 400 bp were extracted from a gel slice using the Qiaquick gel extraction kit (Qiagen) and PCR amplified for 14 cycles using the Illumina primer RP1, and one of the indexed primers (Illumina TruSeq Small RNA kit). The resulting PCR products were purified with Agencourt AMPure Beads XP (Beckman).

### Directional RNA-Seq library

Directional libraries were prepared using the TruSeq Stranded mRNA Sample preparation kit (20020595) following the manufacturer’s instructions (Illumina).

### Library sequencing

Libraries were quantified by fluorimetric measurements with the Qubit® dsDNA HS Assay Kit (ThermoFisher). 51-bp Single Read sequences were generated on the Hiseq2000 sequencer according to manufacturer’s instructions (Illumina). The multiplexing level was 6 or 8 samples per lane. Sequences were demultiplexed using the Illumina pipeline (Gerald, included in CASAVA version 1.7) giving FASTQ formatted reads. The FASTQ formatted reads were cleaned from adapter sequences and sequences of low quality using an in-house program (https://github.com/baj12/clean_ngs). Only sequences with a minimum length of 25 nucleotides were considered for further analysis. Bowtie ([20], version 0.12.7, −-chunkmbs 400, −m 50, −e50, −a - - best, −q) was used to align to the N16961 reference genome (accession numbers AE003852 and AE003853) with modified gene and new ncRNAs positions (this work). HTseq-count ([21], http://htseq.readthedocs.io/, parameters: -m intersection-nonempty, −s yes, −t gene) was used for counting reads associated with transcripts. For TSS analysis only full-length reads (51mers) that aligned to the reference were considered. The first position of each read was extracted in a strand specific manner.

### Statistical analysis of SOS RNA-Seq

Count data were analyzed using R version 3.2.0 [22] and the Bioconductor package DESeq2 version 1.8.1 [23]. Data were normalized with DESeq2 and the “shorth” parameter. The dispersion estimation and statistical test for differential expression were performed with default parameters (including outlier detection and independent filtering). The generalized linear model was set with a single covariate corresponding to the biological condition, biological replicates being included into each condition (SOS vs. noSOS). Raw *p-values* were adjusted for multiple testing according to the Benjamini and Hochberg (BH, [24]) procedure and genes with an adjusted p-value lower than 0.001 were considered differentially expressed.

### SOS operons RT-PCR

RT was performed for 1 h at 55 °C with 5 μg of *V. cholerae* N16961 total RNA, extracted from culture treated with MMC, using the Superscript III (Invitrogen) and primers described in Additional file 2. Superscript III was necessary due to the operon’s length. A control was performed without the enzyme. PCR was performed on cDNA using the Herculase II fusion (Agilent).

### *nrd* operon RT-PCR

RT-PCR was performed with 1 μg of *V. cholerae* N16961 total RNA extracted from culture with MMC using the Access RT-PCR system (Promega) with or without (control) avian myeloblastosis virus (AMV) reverse transcriptase and primers described in Additional file 2. This operon’s length did not merit the use of Superscript III.

### Electrophoresis mobility shift assays (EMSA) with LexA protein

A previously constructed overexpression vector harboring the *V. cholerae* N16961 *lexA* gene was used for LexA protein purification that was performed as previously described [17].

Each *V. cholerae* DNA probe was constructed using two complementary 100 bp synthetic oligonucleotides that were assembled together and cloned into the pGEMT vector (Roche). Once confirmed by sequencing, (DIG)-tagged EMSA probes were obtained by PCR amplification using M13 Forward and DIG-M13 Reverse primers. EMSA experiments were performed as previously described [17] using 100 nM of LexA protein and 40 ng of each DIG-tagged DNA probe. All samples were loaded in 6% non-denaturing Tris-glycine polyacrylamide gels. DIG-labeled DNA-protein complexes were detected following the manufacturer’s protocol (Roche).

### Determination of mitomycin C susceptibility

We assessed the susceptibility to MMC in marine medium according to the method described by Han et al. [25].

### UV survival tests

These assays were performed as previously described [26] using 35 J/m2 UV irradiation.

### P_lac_-*cep* insertion inside specific Tn7 site in N16961 *V. cholerae* genome

*V. cholerae cep* under control of the P_lac_ promoter was inserted in the D074 strain as previously described [27].

### ncRNA and TSS content comparisons

The ncRNA and TSS content we identified in *V. cholerae* was compared to the transcripts reported in three previously published studies [12–14]. For ncRNAs, transcripts that matched any of the coordinates found in our screen, regardless of length, were considered. ncRNAs also had to be transcribed in the same strand to be reported. Since some of the former studies did not screen for TSSs, we did not expect the start sites to be identical. Moreover, since the methods utilized in former studies vary considerably in terms of RNA extraction technique and read length, we did not expect to identify identical lengths for ncRNAs. Therefore, any overlap between ncRNAs from the different studies was sufficient for match report. For TSSs, only exact coordinate matches were taken into account and reported.

## Results

### Transcriptional start sites determination

We started our study by performing extensive TSS mapping of the *V. cholerae* gene set. In order to identify the full complement of TSSs for *V. cholerae* genes, we gathered the results obtained from RNA coming from 7 different growth conditions: Marine broth +/− MMC, LB with high NaCl level, minimum media, exponential or stationary phase, aerobic or anaerobic (see materials and methods). This choice was validated as a good compromise, as transcripts were detected for the vast majority of genes, and only 2 genes encoding two hypothetical proteins (VC0507 and VC1404), exhibited no reads in the pooled RNA-Seq experiments. A new protocol for RNA purification was developed to obtain intact RNAs (see materials and methods). In addition, cDNAs were synthesized from RNAs treated with or without Tobacco Acid Pyrophosphatase (TAP), and with both Terminator 5’-Phosphate-Dependant Exonuclease (TEX) and TAP to identify genuine TSSs. TAP treatment allowed for the detection of native 5’ends of RNAs. While primary transcripts with 5′-triphosphate ends, which survive TEX treatment, were enriched in the TEX+ libraries. We combined two independent sequencing approaches – directional whole-transcript cDNA sequencing (RNA-seq) and differential 5’end sequencing.

To ensure TSS annotation accuracy, manual curation of the *V. cholerae* N16961 genome was performed using the MAGE platform of the Genoscope [28, 29] (www.genoscope.cns.fr/agc/microscope/home/index.php; Genome Browser *Vibrio cholerae* N16961).

All TSSs were manually validated when a corresponding peak was found with TEX and/or TAP treatment fewer than 50 bases from the beginning of the reads obtained through RNA-Seq. The TSS also had to show at least 3 bases identical to the 6 bases of the − 10 promoter consensus of its sigma-70 element (TATAAT), located around 10 bases upstream TSS candidates. However, if there were fewer than 3/6 identical bases, but all the other criteria were fulfilled, the TSS was still validated, as *V. cholerae* uses different promoter types, such as those recognized by Sigma-54 [30]. We were able to annotate 3078 TSSs on the *Vibrio cholerae* genome (Additional file 3: Fig. 1a) (www.genoscope.cns.fr/agc/microscope/home/index.php; Genome Browser *Vibrio cholerae* N16961). This led us to change the start codons of 151 genes. The aforementioned modifications took place when TSSs were located inside the predicted coding sequence and upstream from an alternate initiation codon proposed by start prediction methods included in MicroScope platform [28, 29].

**Fig. 1:**
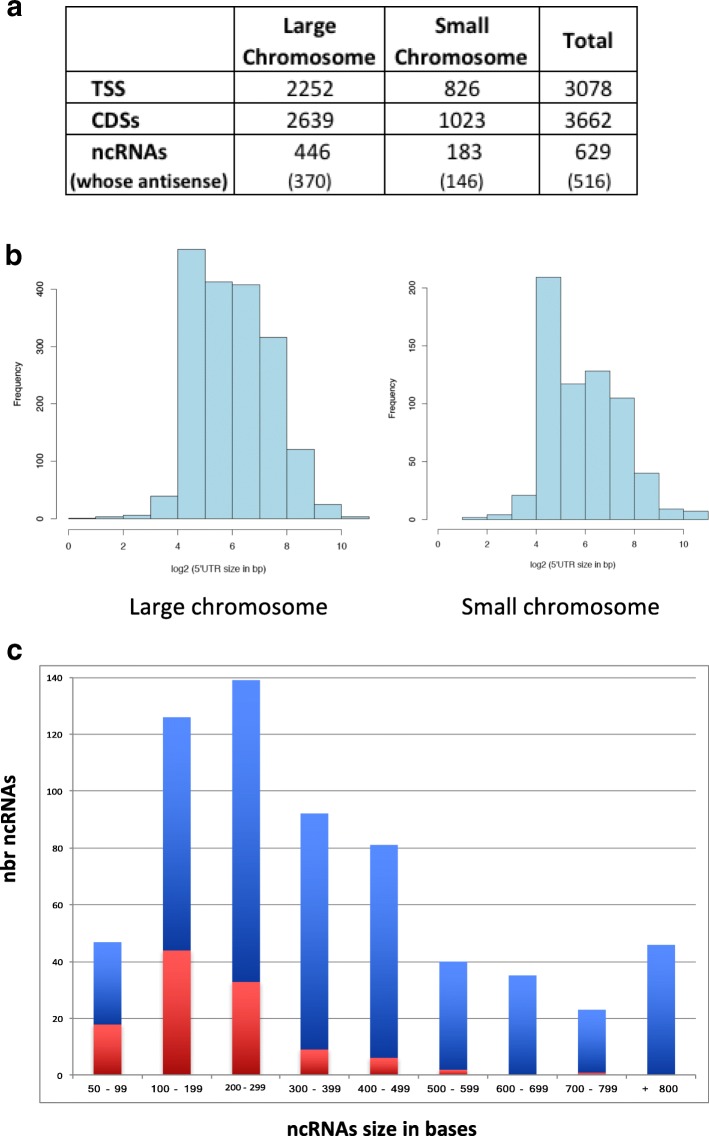
TSS and non-coding RNAs in *V. cholerae*. **a**. TSS, CDS and ncRNA content in the two chromosomes. **b**. 5’UTR sizes in the two chromosomes. **c**. Non-coding RNA sizes. In blue: antisense RNAs, in red: IGR RNAs

The average distance between TSSs and initiation codons (5’UTR), was 116 nt, however the peak of distance distribution was between 16 and 64 nt for both chromosomes (Fig. 1b). There are a number of TSSs located far from the closest validated ATG, opening the possibility for regulatory processes involving these 5’UTR sequences, based on regulator and ncRNA interactions, or riboswitches [31, 32]. The longest 5’UTR sequence (1403 nt) corresponds to an internal promoter inside *priA*. We do not know whether it only allows for transcription of the contiguous genes, as found in *Bacillus subtilis* [33], or drives the expression of a truncated *priA* gene.

### The set of non-coding RNAs in *V. cholerae*

Our analyses also allowed for identification of the full set of native ncRNAs. ncRNAs were reported when a TSS was identified at the 5’end of ncRNA candidates found through RNA-seq. In total, we validated 629 ncRNAs in the *V. cholerae* genome, of which 516 correspond to potential *cis*-antisense RNAs (Fig. 1c) (Additional file 3 and Additional file 4) (www.genoscope.cns.fr/agc/microscope/home/index.php; Genome Browser *Vibrio cholerae* N16961).

ncRNA sizes ranged from 50 to 1907 bases, with antisense RNAs ranging between 100 and 499 bases and a 50 to 300 range for all other ncRNAs (Fig. 1c). The majority of the longest (under 500 bps) ncRNAs were antisense, while the shortest size RNAs corresponded to intergenic region RNAs (IGRs).

We detected most of the previously functionally characterized *V. cholerae* ncRNAs (Additional file 4). However, there were three notable exceptions: *tarA* [34], *qrr1* and *qrr3* [35], for which we were unable to identify any TSS.

We identified ncRNAs belonging to known functional classes using blastn against the Rfam database (http://rfam.xfam.org). This allowed for identification of the ubiquitous tmRNAs (ncRNA154 and 155), those involved in the SRP ribonucleoprotein complex (ncRNA183, 184, 185), RNaseP class A (ncRNA383), the 6S/SsrS RNA (ncRNA392), the amino acid transport regulator *gcvB* (ncRNA161), the leucine operon leader (ncRNA394) and the thiamine pyrophosphate (TPP) (ncRNA215), lysine (ncRNA68) and glycine (ncRNA235) riboswitches (Additional file 4).

Determination of the TSS and ncRNAs content of the *V. cholerae* N16961 genome enabled us to fully characterize the SOS response from a transcriptional standpoint.

### Transcriptional profiling of *V. cholerae* during mitomycin C treatment

Our recent work has emphasized the role played by SOS induction for the response mounted against antibiotic treatment and for integron cassette dynamics in *V. cholerae* [3, 4, 17]. Thus, we were interested in determining the genes that are directly and indirectly transcriptionally regulated in this context. In order to achieve this, we performed comparative RNA-Seq for strain N16961 treated or not with 0.2 μg.mL^− 1^ of MMC, as previously described [17]. MMC is commonly used as a SOS inducer, for it cross-links DNA strands, leading to double strand break formation [17]. Strand-specific RNA-Seq experiments were analyzed and statistical significance was established at an adjusted *p-value* < 0.001, after normalization (see Materials and Methods). 737 genes and 28 ncRNAs were found to be significantly induced more than 2-fold in presence of MMC (Additional files 5 and 6), while 231 genes and 17 ncRNAs were repressed (Additional files 7 and 8). We focused our study on the genes and ncRNAs that were induced more than 2-fold during treatment. Although genes in a variety of categories showed altered transcription during MMC treatment, as seen on Additional file 5, our main focus remains the SOS regulon and its newly identified members.

### Extending the SOS regulon of *V. cholerae*

Erill and collaborators [5] have reported the inventory of genes belonging to the SOS regulon through bioinformatic analysis of LexA binding box distribution, together with validation of LexA binding in vitro. This regulon includes the regulator LexA, the major repressor of SOS regulon, and its associated targets: as RecA and RecX (proteins involved in LexA modulation) RecN, and UvrA (involved in DNA repair) and IntIA and RstAB (involved in integration) (Table 1 top). Transcription of all the genes belonging to the SOS regulon of *V. cholerae,* i.e. those directly controlled by LexA binding [5, 36], was increased at least 2-fold, except for *lon,* which was still slightly induced (1.5-fold) (Table 1 top). Moreover, transcription of the two *rstR* repressors was decreased. This is in agreement with the observed induction of their specific targets, *rstA* and *rstB*, confirming previous results [37] (Table 1 top).

**Table 1:** Genes of the SOS regulon

Label	Gene	Product	Product Type	Role	BioProcess	Ratio +/- MMC
Known SOS regulon genes						
VC0082	rmuC	DNA recombination protein rmuC	cp : cell process	2.1.3 : DNA recombination; 5.8 : SOS response ;	8.1 : DNA replication, recombination, and repair ;	19.7
VC0092	lexA	LexA repressor	r : regulator	2.1.4 : DNA repair ; 2.2.2 : Transcription related ; 3.1.2.3 : Repressor ; 3.3.2 : Regulon (a network of operons encoding related functions) ; 5.8 : SOS response ; 7.1 : Cytoplasm ;	8.1 : DNA replication, recombination, and repair ; 9 : Transcription ; 12.1 : DNA interactions ; 15.10 : Adaptations to atypical conditions ;	25.5
VC0190	uvrD	DNA helicase II	e : enzyme	2.1.1 : DNA replication ; 2.1.4 : DNA repair ; 5.8 : SOS response ; 7.1 : Cytoplasm ;	8.1 : DNA replication, recombination, and repair ; 15.10 : Adaptations to atypical conditions ;	9.1
VC0196	recQ	ATP-dependent DNA helicase recQ	e : enzyme	2.1.1 : DNA replication ; 2.1.3 : DNA recombination ; 2.1.4 : DNA repair; 5.8 : SOS response ; 7.1 : Cytoplasm ;	8 : DNA metabolism ;	2.4
VC0394	uvrA	UvrABC system protein A	e : enzyme	2.1.4 : DNA repair ; 5.6.1 : Radiation ; 5.8 : SOS response ; 7.1 : Cytoplasm ;	8.1 : DNA replication, recombination, and repair ; 15.10 : Adaptations to atypical conditions ;	9.9
VC0397	ssb, exrB, lexC	Single-stranded DNA-binding protein	cp : cell process	2.1.3 : DNA recombination ; 5.8 : SOS response ; 7.1 : Cytoplasm ;	8.1 : DNA replication, recombination, and repair ; 15.10 : Adaptations to atypical conditions ;	3.8
VC0543	recA	Protein recA	e : enzyme	2.1.3 : DNA recombination ; 2.1.4 : DNA repair ; 2.3.6 : Turnover, degradation ; 3.1.3.4 : Proteases, cleavage of compounds ; 5.8 : SOS response ; 7.1 : Cytoplasm ;	8.1 : DNA replication, recombination, and repair ; 11.4 : Degradation of proteins, peptides, and glycopeptides ; 12.3 : Protein interactions ; 15.10 : Adaptations to atypical conditions ;	16.4
VC0544	recX	Regulatory protein recX	r : regulator	Modulates recA activity; 5.8 : SOS response ;	15.10 : Adaptations to atypical conditions ;	11.4
VC0852	recN, radB	DNA repair protein recN	cp : cell process	2.1.3 : DNA recombination ; 2.1.4 : DNA repair ; 5.8 : SOS response ; 7.1 : Cytoplasm ;	8.1 : DNA replication, recombination, and repair ; 15.10 : Adaptations to atypical conditions ;	47.0
VC1191	unfA	putative Superfamily II DNA and RNA helicase	pe : putative enzyme	; 5.8 : SOS response ;		32.7
VC1368	unfB	conserved hypothetical protein	o : ORF of unknown function	; 5.8 : SOS response ;		16.0
VC1453	rstB1	RstB phage-related integrase	e : enzyme	8.1.4 : Integration, recombination ; 5.8 : SOS response ;	17.2 : Prophage functions ;	25.7
VC1454	rstA1	RstA phage-related replication protein	h : extrachromosomal origin	8.1.2 : Replication; 5.8 : SOS response ;;	17.2 : Prophage functions ;	33.8
VC1455	rstR1	Cryptic phage CTXphi transcriptional repressor rstR	r : regulator	3.1.2.3 : Repressor ; 7.1 : Cytoplasm ; 8.1.3 : Regulation ; 5.8 : SOS response ;	12 : Regulatory functions ; 17.2 : Prophage functions ;	0.3
VC1462	rstB2	RstB phage-related integrase	e : enzyme	8.1.4 : Integration, recombination; 5.8 : SOS response ;	17.2 : Prophage functions ;	28.2
VC1463	rstA2	RstA phage-related replication protein	h : extrachromosomal origin	8.1.2 : Replication; 5.8 : SOS response ;	17.2 : Prophage functions ;	33.7
VC1464	rstR2	Cryptic phage CTXphi transcriptional repressor rstR	r : regulator	3.1.2.3 : Repressor ; 7.1 : Cytoplasm ; 8.1.3 : Regulation; 5.8 : SOS response ;	12 : Regulatory functions ; 17.2 : Prophage functions ;	0.3
VC1845	ruvB	Holliday junction ATP-dependent DNA helicase ruvB	e : enzyme	2.1.3 : DNA recombination ; 2.1.4 : DNA repair ; 5.8 : SOS response ; 7.1 : Cytoplasm ;	8.1 : DNA replication, recombination, and repair ; 15.10 : Adaptations to atypical conditions ;	4.1
VC1846	ruvA	Holliday junction ATP-dependent DNA helicase ruvA	e : enzyme	2.1.3 : DNA recombination ; 2.1.4 : DNA repair ; 5.8 : SOS response ; 7.1 : Cytoplasm ;	8.1 : DNA replication, recombination, and repair ; 15.10 : Adaptations to atypical conditions ;	4.7
VC2043	topB	DNA topoisomerase 3	e : enzyme	2.1.1 : DNA replication ; 2.2.2 : Transcription related ; 3.1.1.1 : DNA bending, supercoiling, inversion ; 7.1 : Cytoplasm; 5.8 : SOS response ;	8.1 : DNA replication, recombination, and repair ; 9 : Transcription ; 12 : Regulatory functions ;	4.8
VC2287	dinB	DNA polymerase IV	e : enzyme	2.1.1 : DNA replication; 5.8 : SOS response ;	8.1 : DNA replication, recombination, and repair ;	19.4
VC2326	yebG	dsDNA-binding SOS-regulon protein	pcp : putative cell process	; 5.8 : SOS response ;		4.0
VC2711	recG, spoV, radC	ATP-dependent DNA helicase recG	e : enzyme	2.1.1 : DNA replication; 5.8 : SOS response ;	8 : DNA metabolism ;	4.1
VCA0291	intIA	Site-specific recombinase IntIA	e : enzyme	2.1.3 : DNA recombination ; 7.1 : Cytoplasm ; 8.1.4 : Integration, recombination; 5.8 : SOS response ;	8.1 : DNA replication, recombination, and repair ;	14.4
VCA0952	vpsT, csgD	LuxR family transcriptional regulator VpsT	r : regulator	3 : Regulation ; 3.1.2 : Transcriptional level ; 5.12 : Biofilm production ; 5.8 : SOS response ;	12 : Regulatory functions ; 12.1 : DNA interactions ; 14 : Cell envelope ; 14.1 : Surface structures ; 14.3 : Biosynthesis and degradation of surface polysaccharides and lipopolysaccharides ;	2.8
New SOS regulon genes						
VC0083	ubiE	Ubiquinone/menaquinone biosynthesis methyltransferase UbiE	e : enzyme	1.5.3.11 : Menaquinone (MK), ubiquinone (Q) ;	4.5 : Menaquinone and ubiquinone ;	9.0
VC0084	ubiJ	Ubiquinone biosynthesis protein UbiJ	e : enzyme	1.5.3.11 : Menaquinone (MK), ubiquinone (Q) ;	4.5 : Menaquinone and ubiquinone ;	5.2
VC0085	ubiB	ubiquinone biosynthesis protein UbiB	e : enzyme	1.5.3.11 : Menaquinone (MK), ubiquinone (Q) ; 5 : Inner membrane protein	4.5 : Menaquinone and ubiquinone ;	3.7
VC0086	tatA	Sec-independent protein translocase TatA	t : transporter	4.2.A.64 : The Type V Secretory Pathway or Twin Arginine Targeting (Tat) Family ; 4.S.160 : protein ;	7 : Transport and binding proteins ;	3.2
VC0087	tatB	Sec-independent protein translocase protein TatB	t : transporter	4.2.A.64 : The Type V Secretory Pathway or Twin Arginine Targeting (Tat) Family ; 4.S.160 : protein ;	7 : Transport and binding proteins ;	4.0
VC0088	tatC	Sec-independent protein translocase protein TatC	t : transporter	4.2.A.64 : The Type V Secretory Pathway or Twin Arginine Targeting (Tat) Family ; 4.S.160 : protein ; 5 : Inner membrane protein	7 : Transport and binding proteins ;	5.5
VC0091		putative O-Methyltransferase involved in polyketide biosynthesis	pe : putative enzyme	5.6.4 : Drug resistance/sensitivity ;	15.8 : Toxin production and resistance ;	13.7
VC0851	smpA	small protein A	lp : lipoprotein			12.7
VC1190		putative phosphoribosylaminoimidazole-succinocarboxamide synthase PurC	pe : putative enzyme	1.5.2.1 : Purine biosynthesis ; 2 : Cytoplasmic	2.3 : Purine ribonucleotide biosynthesis ;	16.8
VC1369		putative ABC-type nitrate/sulfonate/bicarbonate transport systems, periplasmic component	pt : putative transporter	4.3.A.1.p : periplasmic binding component; 9 : Periplasmic	7 : Transport and binding proteins ;	2.4
VC1370		putative Signal transduction histidine kinase domain, Methyl-accepting chemotaxis domain and GGDEF family protein	pr : putative regulator	3 : Regulation ; 5 : Inner membrane protein	12 : Regulatory functions ;	2.3
VC1461	cep	Colonization factor	f : factor	5.13 : Virulence associated ; 5 : Inner membrane protein	15.9 : Pathogenesis ;	52.4

The different categories correspond to those of MAGE annotation platform

**Fig. 2:**
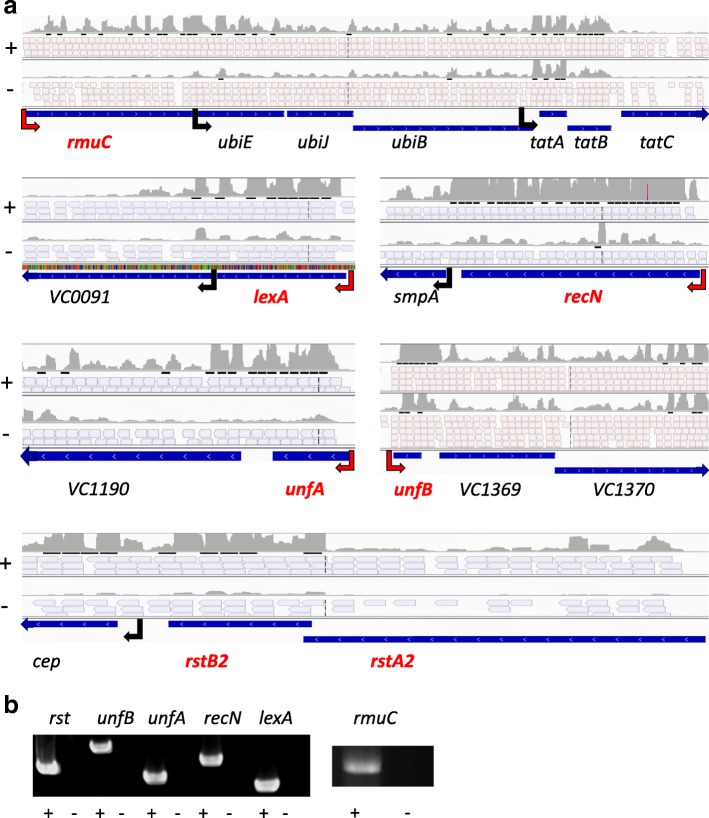
SOS regulon operons. **a**. RNA-seq data in marine medium in presence (+) or absence of mitomycin C (−). For each operon, the top track corresponds to the piling up signal of all reads. The middle tracks correspond to individual reads; for pink reads: the sense of transcription is leading strand; in blue: the sense of transcription is the lagging strand. The bottom track shows gene annotation in the *Vibrio cholerae* genome. Genes known to belong to the SOS regulon are shown in red. Black arrows correspond to the intermediary transcriptional starts, red to the gene’s first TSS. **b**. RT-PCR for entire operons in presence (+) and absence (−) of superscript III reverse transcriptase

Among the genes whose expression was increased in presence of MMC, several are located nearby and in the same orientation as previously known SOS regulon genes. RNA-Seq data showed that 12 of them seemed to be transcribed together with the neighboring SOS regulon genes, even if most of them possess their own independently regulated promoter (Fig. 2a). To demonstrate their co-transcription, RT-PCRs were performed along the entire transcript. In all cases, we obtained a fragment corresponding to the full-length transcript (Fig. 2b). This demonstrates that the *ubiEJB* operon*,* the *tatABC* operon, *VC0091*, *smpA*, *VC1190*, *VC1369–1370* and *cep* are co-induced with their adjacent SOS regulon genes when this response is triggered. Thus, they should be included in the *V. cholerae* SOS regulon (Table 1 bottom).

### Two major pathways are induced by mitomycin C beyond the SOS regulon

Genes induced at least two-fold by MMC treatment belonged to a variety of functional categories. Overall, in addition to those that are part of the SOS response (with the exception of *lon*), we saw an increase in expression for many genes involved in oxidative stress response and nucleotide metabolism (Additional file 5).

MMC treatment has a dual effect as it impairs DNA directly by DNA strand cross-linking and indirectly through DNA oxidation, as MMC reacts with molecular oxygen to produce reactive semiquinone and superoxide [38]. We saw a clear induction of many genes involved in oxidative stress response (*gor*, *sodB*, *fabA*, *zwf*, *katB*, genes included in the Fe-S and thiol-redox systems, and related to synthesis of cysteine, an antioxidant amino acid [39–41]) (green lettering in Additional file 5), and of those implicated in oxidized base repair (*mutY*) [42, 43].

**Fig. 3:**
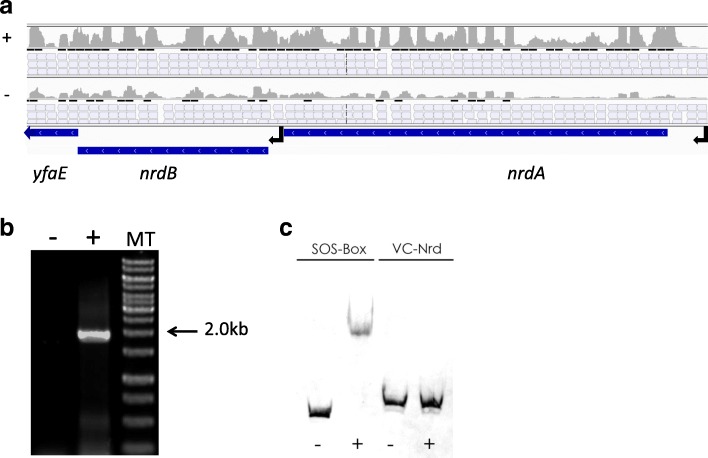
*nrdAB*-VC1254 expression. **a**. Marine RNA-seq data in presence (+) or absence of mitomycin C (−). The top tracks correspond to the piling up signal of all reads. The middle tracks correspond to individual reads. Arrow corresponds to transcriptional start. The bottom track shows the gene annotation of *Vibrio cholerae*. **b**. RT-PCR of the entire operon in presence (+) and absence (−) of AMV reverse transcriptase. MT corresponds to 1Kb ladder. **c**. EMSA performed using *nrdAB* promoter region (VC-Nrd) in presence (+) and absence (−) of LexA protein. A positive SOS-box probe was included as a control

Fifty-five induced genes are directly or indirectly involved in nucleotides biosynthesis, interconversion, transport and salvage (blue lettering in Additional file 5), consistent with the need to repair MMC inflicted DNA damage, as previously found in *E. coli* [44]. Among these genes, four encode ribonuclease reductases involved in deoxyribonucleotide biosynthesis organized in two operons: *nrdD* and *nrdG* (*VCA0511–512*) and *nrdA* and *nrdB* (*VC1256–1255*). The aforementioned genes showed almost the same expression increase, and although they are organized as an operon, we identified a TSS upstream from *nrdA* and *nrdB* (Fig. 3a), showing a similar organization and regulation to that observed in *E. coli* [45]. Downstream of *nrdB* and induced similarly to *nrdA* and *nrdB*, is located a putative ferredoxin gene*, VC1254*. This syntenic organization is conserved in many bacteria, including *E. coli*, where the product of the orthologous gene, *yfaE* (Fig. 3a), is involved in the NrdAB ribonuclease diferric-tyrosyl radical maintenance [46], showing a functional link with NrdA and B, though their co-transcription had not been established. To determine whether *VC1254 (yfaE)* is co-transcribed with *nrdAB,* we performed a semi-quantitative RT-PCR using primers hybridizing with the 5′ extremity of *nrdA* and the 3′ end of *VC1254 (yfaE)*, and we were able to amplify the corresponding 1.9 kb fragment (Fig. 3b), demonstrating that *VC1254 (yfaE)* belongs to the *nrdAB* operon. Since a sequence related to the LexA binding box (CTGTATATATATACAG) was present near the *nrdA* promoter, we performed EMSA with purified LexA protein and the *nrdA* promoter region (Fig. 3c). No LexA binding was detected, showing that the *nrdAB-VC1254 (yfaE)* operon is not a part of the SOS regulon, similarly to what has been reported in *E. coli* [47].

### The set of ncRNAs induced by mitomycin C

We found that among the identified 629 ncRNAs, 28 showed an increase in abundance of at least 2 fold after MMC treatment. Eighteen of these were located in IGRs and have known functions, while ten were previously uncharacterized, out of which nine are IGRs and one was transcribed in antisense. Several previously described ncRNAs are known to be involved in DNA repair or are regulated by the SOS response (*tmRNA, RNP, ryhB*), and our results confirmed this. tmRNA, which is involved in mRNA processing, has been implicated in DNA-protein crosslink repair in *E. coli* [42]. The Rnase P (RNP) class A (M1 RNA), which is also involved in mRNA processing, inhibits gyrase A providing a higher quantity of RecA in *E. coli* for full SOS response induction [48]. Moreover, the bacterial signal recognition particle (SRP), which is known to regulate the two aforementioned IGRs [49], is also impacted. *csrB* and *csrC* upregulation is contrasted with the reported repression during SOS response induction in *E. coli* [50], suggesting a different role for these ncRNAs in *V. cholerae*. The induction of *ryhB*, which is involved in iron metabolism and antioxidant defense, can be connected to the oxidative stress arising from MMC treatment. Consequently its 27 target genes (e.g. the enterobactin receptor, fumarate reductase, cytochrome) characterized in *V. cholerae* also underwent transcriptional variation [7, 8].

In addition, several characterized ncRNAs, without any known link to DNA repair, SOS response and oxidative stress were found to be regulated. This included *vqmR*, a regulator of biofilm formation in *V. cholerae,* and three of its targets [14]; *mtlS* and its mannitol PTS utilization operon target [51, 52]; *gcvB*, involved in regulation of several pathways in *E. coli*, such as amino acid metabolism and transport, the two-component system PhoP/PhoQ [53–57]; and *micX*, a regulator of the outer membrane protein (OMP) [51, 52]. However, the impact of *micX* increase on its known targets in *V. cholerae* [51, 52] was very low or inexistent (less than two-fold).

### Phenotypic characterization of new members of the SOS regulon and the most highly induced genes and ncRNAs by MMC treatment

Our goal here was to further characterize the SOS response, and evaluate the phenotypic contribution, in terms of resistance to genotoxic stress, of the new members of the SOS regulon uncovered in this study and *unfA* and *unfB*, whose potential role in DNA repair remains to be determined, as well as the most highly induced (more than 5-fold) genes. Some genes with over 5-fold induction were impossible to delete and therefore were not further studied as they are likely essential under the applied conditions (VCA0862, VC0940, VCA1063, VIBCH10366, VC0059). We focused our exploration on twenty-five genes (list of strains with deletions for these genes found in Additional file 1). For the SOS regulon gene set, we only studied genes without an obvious role in DNA repair or protection. We excluded *VC1190* that encodes a putative Phosphoribosylaminoimidazole-succinocarboxamide synthase (implicated in purine metabolism for de novo nucleotide synthesis necessary to DNA repair) and the *ubiEJB* operon that encodes enzymes used in ubiquinone (coenzyme Q10) biosynthesis, known to be required for stress-induced mutagenesis, presumably via activation of RpoS-response [58–60]. In addition, among the ncRNAs, we chose 5 candidates with known functions (*csrB, csrC, gcvB, ryhB* and *vqmR*), which could possibly play a role in the response to genotoxic agents and 5 ncRNAs of unknown function that exhibited high level of expression or induction by MMC (ncRNA20, 59, 398, 547 and 586). All these genes/ncRNAs were inactivated (list of strains with deletions found in Additional file 1) and the corresponding mutants were phenotypically assayed.

**Fig. 4:**
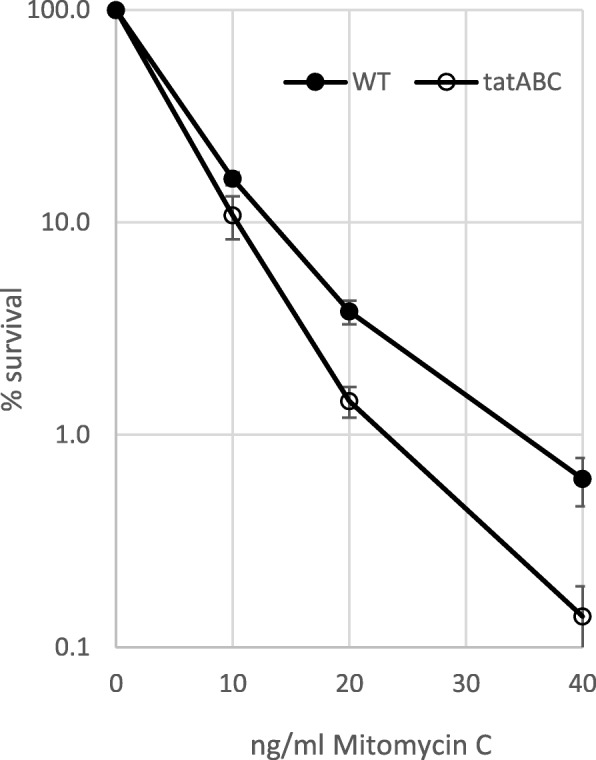
Susceptibility to MMC of *tatABC* deficient strain. Marine culture with wild-type or mutant strains were treated with different concentrations of MMC for two hours and the relative survival on plate, in comparison to wild-type, was calculated. Error bars portray the standard deviation. Survival rates were found to be statistically significant (*p* < 0,05)

First, we explored MMC resistance to define genes/ncRNAs specifically involved in this process. Among these mutants, the *tatABC* operon deleted strain was the only one that exhibited significantly higher (*p* < 0,05) MMC susceptibility when compared to the wild type strain (Fig. 4), demonstrating its role in overcoming this stress.

**Fig. 5:**
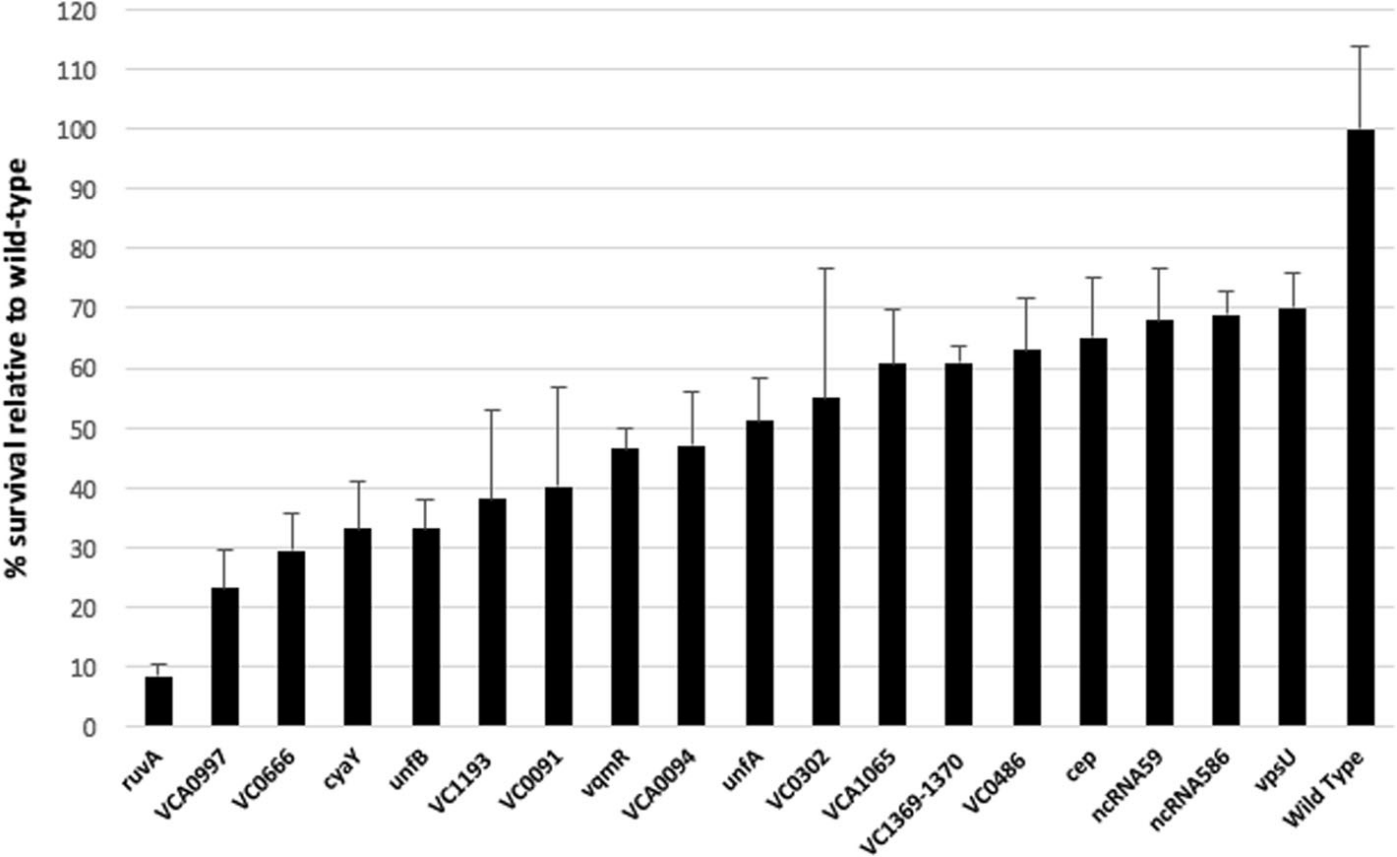
Susceptibility to UV. Marine culture with wild-type or mutant strains were UV irradiated with 35 J/m^2^, and the relative survive on plate, in comparison to wild-type, was calculated. *ruvA* mutant is a control

To determine if they could possibly be more broadly involved in DNA repair or protective processes, all mutant strains were assayed for their UV susceptibility. Indeed, UV irradiation leads to DNA damages and SOS response induction, as shown in many bacteria including *Vibrio* species (e.g. *Vibrio parahaemolyticus)* [61, 62]. Moreover, UV treatment leads to mostly single strand DNA damage due to bipyrimidine dimers, producing mutagenic and cytotoxic DNA lesions and dsDNA breaks. The aforementioned lesions are thus different from those resulting from MMC treatment, which is a DNA strand cross-linking agent, causing O6-guanine alkylation, DNA oxidation and ultimately dsDNA breaks. A *ruvA* deficient strain was used as control, as it is known to be highly sensitive to UV [63]. Among mutants assayed, only 17 exhibited a reduced survival to UV irradiation (Fig. 5), suggesting their involvement in the response to this stress, and more generally in DNA repair or protection. Among the newly identified SOS genes, we found that 6 of these (*unfA*, *unfB*, *VC0091*, *VC1369–1370* and *cep*) are involved in UV resistance (Fig. 5), supporting their inclusion in this specific response’s regulon. Mutants of three ncRNAs (ncRNA59, ncRNA586 and *vqmR*) (Fig. 5) were also found to have a reduced viability toward UV. To our knowledge, these are the first ncRNAs ever implied in survival to this genotoxic stress.

Seven ncRNAS (*csrB, csrC, gcvB, ryhB*, ncRNA20, 398 and 547) and seven genes (*rtxH, smpA*, VC0025, VC0424, VC0484, VC1272 and VC2115) highly expressed and/or induced by MMC treatment were not found to be involved in UV and MMC resistance.

Among the 17 genes shown to play a role in UV resistance, *cep* is the only one encoded in a prophage (CTX phage). Cep is a small protein exported through the inner membrane, which is involved in intestine colonization [64]. In order to confirm its phenotypic role in UV resistance, we tested whether its overexpression would increase bacterial resistance towards this stress. We ectopically inserted *cep,* under the control of a Plac promoter, inside a mini-Tn7 at the chromosomal *attTn7* site in a *cep* deficient strain. This complementation restored UV resistance, even at a higher rate than the wild-type (data not shown), confirming the role of Cep in this process.

Analysis of the regions around the promoter of the genes and ncRNAs found to play a role in UV resistance and not included in the previously defined SOS regulon [5], revealed the presence of a sequence related to the LexA box consensus for several of them. In order to establish if they were part of the SOS regulon, EMSA were performed on these DNA sequences with purified *V. cholerae* LexA protein. No band shift was observed, even at high LexA concentration, for any of these candidates (Additional file 9). This showed that *VC0486, VC0302, VCA0997, VC0123, VC0916, VC1193, VCA0094* and the ncRNA59 are not part of the canonical SOS regulon.

### Comparative analyses with previously published ncRNA/TSS screens

Comparison with 3 previously published studies that have made attempts at characterizing the ncRNA content in *V. cholerae* yielded a shockingly low number of shared transcripts. Out of the thousands/hundreds of ncRNA candidates identified by all 4 studies (ours, Liu’s, Raabe’s and Papenfort’s) only 10 were identified in all of them (Additional file 10). Two out of the three previously published screens use massively parallel sequencing technologies: Liu’s and Papenfort’s [12, 14], and are therefore in principle more likely to show a higher degree of overlap. This comparison yielded a total of 34 common ncRNAs. This doesn’t include all functionally characterized ncRNAs, highlighting the disparate outcomes produced by different studies. Comparison of our TSS analysis with Papenfort’s study, which also focused on TSS detection identified 1853 out of 3078 start sites in common (Additional file 11). This is an acceptable level of concordance with the previously published screen, with slightly over 60% of the start sites having an exact match in both studies. The difference in total number of identified TSSs could reflect the differences in the growth conditions taken into account, and the relatively arbitrary rules used to determine the presence of a TSS at a given position.

## Discussion

Our objective was to fully characterize the SOS regulon as well as novel genes related to this response during genotoxic stress in *V. cholerae*. For this, we used a transcriptomic screen of cells treated with subinhibitory concentration of MMC [17].

We first focused our analysis on the effect of MMC on the *V. cholerae* transcriptome. RNA-Seq experiments on *V. cholerae* culture in presence of MMC show that 20.1% of its genes are induced at least 2 fold, a figure similar to what has been found in previous transcriptomic studies performed in other proteobacteria such as *Pseudomonas aeruginosa*, *Acinetobacter* and *E. coli* under similar stresses [44, 65, 66].

Among the induced genes, we found most of the previously known members of the SOS regulon [5], validating our choice for MMC as an SOS inducer. Moreover, we demonstrated the functional involvement of UnfA and UnfB in the SOS response (Fig. 5). Previously, their SOS involvement was only determined through LexA binding to their promoter regions [5]. Here, we show that they are induced during the SOS response and that they seem to participate in DNA repair/protection processes, as deficient strains were more susceptible to UV irradiation.

Our study also demonstrates that 12 genes, adjacent to previously identified SOS regulon genes are transcribed in operon with the latter and thus showed an expression increase in presence of MMC (Fig. 2). This extends the *V. cholerae* SOS regulon to 37 genes (Table 1). Among these 12 new SOS genes, 4 can be linked directly to DNA damage salvage. This includes the *ubiEJB* operon, which allows for ubiquinone (coenzyme Q10) biosynthesis, which in turn reduces DNA damage and participates in DNA breaks repair [58–60]. Another newly included SOS gene, *VC1190* encodes for a putative Phosphoribosylaminoimidazole-succinocarboxamide synthase, involved in de novo nucleotide synthesis, which is necessary to DNA repair. The function of other SOS co-expressed genes is less obvious. However, phenotypic assays using deficient strains shed light on their role. Indeed, mutants for *VC0091* (putative O-Methyltransferase), *VC1369* and *VC1370* (membrane proteins putatively involved in transport and regulation respectively) or *cep* (a small colonization factor located in inner membrane and previously involved in intestine colonization [67]) were less resistant to UV irradiation, similarly to the *unfA* and *unfB* mutants (Fig. 5). These genes may also have a general role in DNA repair or protection processes since UV treatment leads to mutagenic and cytotoxic DNA lesions and dsDNA breaks, which are different from damages resulting from MMC treatment (DNA strand cross-linking, O6-guanine alkylation, and ultimately DNA oxidation and dsDNA breaks).

In addition, we found that the *tatABC* operon deletion had no impact on UV irradiation resistance, but reduced 4-fold the survival rate after incubation with 40 ng/ml MMC. This operon encodes for proteins necessary to the type V secretory pathway, known to help *V. cholerae* survive in the environment, probably by favoring excretion of xenobiotic elements [68], as it confers protamine resistance in *E. coli* [69]. This suggests that SOS regulon members can also reduce DNA damages by eliminating their source.

The last of these newly included genes, *smpA*, encodes a lipoprotein, whose orthologue in *S. typhimurium* plays a role in cell envelope integrity and virulence [70], has no effect on UV radiation or MMC resistance. Consequently, its role in SOS induction remains to be established.

The synteny between these genes and their neighboring SOS genes is conserved in all *Vibrio* species, except for the *unfA* and the *rstAB2* associated operons. The *recN-smpA* and *rmuC-tatABC* genomic organization is also conserved in other proteobacteria such as *E. coli* and *S. typhimurium*, supporting a selective advantage for this co-regulated organization and their inclusion in these species’ SOS regulon. It is interesting to note that expression of two proteins, RdgC and the ATP synthase (*atpCDGAHFEBI*), that are not members of the SOS regulon, but are known to impact RecA function, is induced. RdgC plays a modulator role for RecA in *V. cholerae*, by preventing RecA binding, to avoid replication fork progression problems and allow PriA binding to promote replication restart [71]. Furthermore, ATP is essential for the activation of RecA, and its ability to form nucleoprotein filaments [72]. Such an increase in ATP concentration has previously been observed in *E. coli* after SOS induction [73]. In addition, transcription of four toxin/antitoxin (TA) systems (the three ParDE and the toxin HigBA2), located in the superintegron cassette array [74] was found to be increased, even though their promoters are not directly controlled by LexA. Since ParE-mediated DNA damage through gyrase inhibition [75] is known to activate SOS response in *V. cholerae* [76], its transcriptional increase could play an amplifying role for this response in these conditions.

Moreover, a total of 55 induced genes are directly or indirectly involved in nucleotides biosynthesis, interconversion, transport and salvage (blue lettering in additional file 5), consistent with the need for nucleotides to repair DNA damages caused by MMC, as previously found in *E. coli* [44]. Additionally, the DNA binding protein Fis (required for mutagenic break repair in *E. coli* [77]), MutY (A:8-OG mispair Base-excision repair), MutS (O(6)-methylguanine damage and mismatch repair), and both MnmE and MnmG (major role in *azaC* induced tmRNA tagging, allowing tolerance/repair to DNA-protein crosslinks in *E. coli* and *V. cholerae* [42, 43]) are also induced. These last proteins, as well as RecN [78], the putative Universal stress protein UspA [79] and thiamine biosynthesis and transport proteins [80, 81] are likewise implicated in oxidative stress resistance [40]. In agreement with the oxidative stress caused by MMC treatment [82], we see a clear induction of many proteins involved in the specific oxidative stress response (Gor, SodB, FabA, Zwf, KatB, thioredoxin and glutaredoxin, proteins of Fe-S and thiol-redox systems and related to synthesis of the antioxidant cysteine amino acid [39], [40, 41], [83]).

In addition to the new SOS regulon members, we characterized 9 proteins also involved in UV resistance, but not directly regulated by LexA. Three were annotated as hypothetical proteins (VC0666, VC1193, VCA1065) and this is their first functional characterization. Several of these nine proteins, such as the iron chaperone CyaY, VC0302 (a putative 3-phenylpropionic acid transporter) and VpsU (involved in *Vibrio* polysaccharide (VPS) production) might participate in protection against environmental stress and could limit new DNA damages. As the VCA0997 hypothetical protein is predicted to be located in the inner membrane, it could also participate in this process, as many other transporters or membrane proteins found in our screen. On the other hand, the effect of VC0486 and VCA0094, which encode putative transcriptional regulators, is likely indirect through a regulatory role on the expression of genes that could play a role against DNA damage. Indeed, additional regulators were found to be induced in our RNA-Seq data (Additional file 5). It is important to mention that several other proteins may also be inhibited/induced due to MMC. This highlights the fact that not all transcriptomic changes provoked by MMC treatment are directly linked to the SOS response.

Finally, we identified 28 ncRNAs that showed an increase in abundance of at least 2-fold after MMC treatment. Two are linked to DNA repair or to SOS response regulation: tmRNA has been implicated in DNA-protein crosslinks repair in *E. coli* [42], and Rnase P class A inhibits gyrase A allowing higher RecA level in *E. coli* for full SOS response induction [48]. Consequently, we detected an induction of the bacterial signal recognition particle (SRP) RNA component, known to regulate these two IGRs [49]. However, no LexA box was detected close to its promoter, preventing its inclusion in the SOS regulon. *ryhB* is involved in iron metabolism and antioxidant defense, which can be connected to the oxidative stress arising from MMC treatment [7, 8]. We also show, for the first time, a role in DNA repair or protective process for several ncRNAs. Indeed, three of the induced ncRNAs, ncRNA59, ncRNA586 and *vqmR* were shown to affect UV resistance when mutated (30–50% viability reduction).

In order to clarify previously published conflicting data on the *V. cholerae* ncRNAs content [12, 13], we decided to define first the whole set of RNA molecules (mRNAs and ncRNAs) that are transcribed by this bacterium, grown in a variety of conditions. Additionally, we used this transcriptomic screen to establish a genome-wide TSS map. For this purpose, a protocol for RNA purification and 5′ end RNA-Seq libraries generation was developed to allow analysis of intact RNA molecules devoid of degradation during these steps. Moreover, to accurately determine TSSs, RNAs were treated by TAP and both TEX+TAP, as performed to define the whole TSS set of *Legionella pneumophila* by Sahr and collaborators [84], as native 5’ends of RNAs are detected with these treatments (TAP increasing these type of ends and TEX decreasing non native 5′ ends). In order to get the deepest coverage possible, experiments were performed from cells grown in seven different conditions. The fact that we detected expression of all but two genes validated our experimental approach. The manual validation of all the data, using transcriptional rules, allowed us to exclude truncated ncRNAS and processed TSSs. This led us to identify 3078 TSS and 629 ncRNAs (additional files 3 and 4). 1853 of the identified start sites have an exact match with the only previously published TSS screen [14]. The discrepancies could be attributed to the different growth conditions used in the two studies, as well as the protocols used and the criteria applied for TSS report, as manual validation for all TSSs was performed in our study. We believe manual curation of TSSs adds value to our screen.

The number of ncRNAs was lower than what is reported in three previously published studies [12–14]. As formerly notified by Toffano-Nioche and collaborators [15], we found that many of their ncRNAs correspond to truncated forms of our characterized ncRNAs. These discrepancies are likely due to differences between used protocols and our ncRNA definition by the presence of a start found through TSS mapping. We also identified 5′ or 3′ ends that differ from those published for several functionally characterized *V. cholerae* ncRNAs. In these studies, 5′ extremities had been frequently characterized with classical 5’ RACE experiments, where the major extremity detected may correspond to a processed form. In our study native extremities were detected. On the other hand, 3′ extremities were generally determined by sequence analysis to define the terminator and not through RNA-Seq, which allows for real transcript visualization. ncRNA size analysis showed that the longest ncRNAs were almost always transcribed in antisense, and lacked an obvious terminator. By contrast, the shorter sizes corresponded to IGRs, in agreement with the majority of previously characterized IGRs (e.g. *csrB, csrC* and *csrD* [6]) which fold into short specific secondary structures and are typically ended by a Rho-independent terminator. We detected all previously functionally characterized *V. cholerae* ncRNAs, except for three: *tarA* [34], *qrr1* and *3* [35]. No TSS could be visualized for the aforementioned transcripts. This could reflect either a high instability of their 5′ extremities or the presence of cleavage sites. Ultimately the drastic differences between screens should be taken as a warning sign, as no strategy seems to be capable of capturing all RNA species. Consequently, it has recently been shown that the number of asRNAs is dependent on the genomic AT content [85]. Additionally, the expression of asRNA at low levels exerts little impact in terms of energy consumption. This suggests that antisense transcription is more pervasive than previously thought. Sophisticated computational methods which integrate secondary structure analyses and functional data have been recently developed and reach detection success rates above 80% but plummet to values around 60% when applied to a different bacterial model [86]. This further supports manual curation of massively processed data for higher accuracy. Consensus in terms of reaching a standardized strategy for ncRNA detection/report should be urgently reached in the scientific community.

## Conclusions

Using an extensive transcriptomic screen which yields long and intact RNAs, we expand the *V. cholerae* SOS regulon by unveiling several new members organized in large operons with well-characterized SOS genes. Two of these operons are conserved in most gram-negative proteobacteria supporting their general role in this stress response. In addition, we characterized several genes and ncRNAs involved in this genotoxic response and show that some of them are associated to DNA repair or protection. Moreover, we observed the induction of several transporters or exporting systems, which could possibly be involved in excretion of xenobiotic substances or prevention of their entry inside bacterial cells. This clearly shows that the bacterial response to genotoxic agents goes far beyond DNA repair to protect cells against DNA damages.

## Additional files

Additional file 1: Strains used in the study. (XLSX 27 kb)

Additional file 2: Primers used in the study. (DOCX 119 kb)

Additional file 3: List of *Vibrio cholerae* N16961 TSS characterized in our study. Each sheet corresponds to one strand for one chromosome. (XLSX 68 kb)

Additional file 4: List of *Vibrio cholerae* N16961 ncRNAs characterized in our study. Each sheet corresponds to one strand for one chromosome. (XLSX 66 kb)

Additional file 5: List of *Vibrio cholerae* N16961 genes induced at least two-fold by Mitomycin C treatment. They are classified depending to their function. (XLSX 97 kb)

Additional file 6: List of *Vibrio cholerae* N16961 ncRNAs induced at least two-fold by Mitomycin C treatment. (XLSX 27 kb)

Additional file 7: List of *Vibrio cholerae* N16961 genes repressed at least two-fold by Mitomycin C treatment. (XLSX 31 kb)

Additional file 8: List of *Vibrio cholerae* N16961 ncRNAs repressed at least two-fold by Mitomycin C treatment. (XLSX 28 kb)

Additional file 9: EMSA performed between LexA protein and various promoter region in presence (+) and absence (−) of LexA protein and with a LexA box DNA control (SOS-Box). (PPTX 40364 kb)

Additional file 10: Comparison between ncRNAs content characterized in our study and three previous works [12–14]. (XLS 170 kb)

Additional file 11: Comparison between TSS content characterized in our study and one previous work [14]. (XLSX 43 kb)

## Abbreviations

5’UTR: 5’ Untranslated Region
AMV: Avian myeloblastosis virus
BH: Benjamini and Hochberg
EMSA: Electrophoresis mobility shift assay
IGR: Intergenic region RNA
MMC: Mitomycin C
ncRNA: non-coding RNA
OMP: Outer membrane protein
RNP: Rnase P
SRA: Sequence Read Archive
SRP: Signal recognition particle
ssDNA: Single-stranded DNA
TAP: Tobacco Acid Pyrophosphatase
TEX: Terminator 5’-Phosphate-Dependent Exonuclease
TSS: Transcription start site
UV: Ultra-violet
