# Supplementary material for: Expansion of the SOS regulon of Vibrio cholerae through extensive transcriptome analysis and experimental validation
Source: BMC Genomics. 2018 May 21;19:373. doi: 10.1186/s12864-018-4716-8 (PMC5963079; doi:10.1186/s12864-018-4716-8)
Supplement: Supplementary file 2 — Primers used in the study. (DOCX 119 kb) [file 12864_2018_4716_MOESM2_ESM.docx]

| **RT-PCR**  **Primer name** | Sequence |
| --- | --- |
| nrdrt5 | AAGCGCGTTTGGCTTTTAGC |
| nrdrt3 | CGCAAATCTCTAACGCGACC |
| rmucrt5 | GCAGCTCACTACCGCGCAGC |
| rmucrt3 | GGGTCTGTTCAACGGAAGACAT |
| lexArt5 | CAAGAAGTGTTTGATCTGATC |
| lexArt3 | GCAATCCGATAGAGCGCACG |
| recNrt5 | CCATTGCGATTGATGCTTTAGG |
| recNrt3 | CCACCATCGTTAAATTTCACG |
| unfArt5 | TCTGGGCGAGACAAACCTGA |
| unfArt3 | GCCCCGTGATCTTTTCAGCA |
| unfBrt5 | GCAAGCGATGAATAACCCTG |
| unfBrt3 | GGTGGGCGCAATACACCGTA |
| rstrt5 | ATGCTTCATTCCGTCACGCG |
| rstrt3 | GCCTTACGAATTAAGCCAATC |

**EMSA Primers:**

**nrd-box-F**

gaataaagcgatgtgcaaacggcgattttttacaatcaattctgctcttagatcatcagtaagtaggtactaactgatttttttaattttgctagttatA

**nrd-box-R**

ataactagcaaaattaaaaaaatcagttagtacctacttactgatgatctaagagcagaattgattgtaaaaaatcgccgtttgcacatcgctttattcA

**VC0302Bx1F**

tgatccgccgattggcaccggtaacttgacggtgaatctgttgatataagcaatcgggaaacgatgagttgcgtaatcttcccttttttagacgcgaatA

**VC0302Bx1R**

attcgcgtctaaaaaagggaagattacgcaactcatcgtttcccgattgcttatatcaacagattcaccgtcaagttaccggtgccaatcggcggatcaA

**VCA0094Bx1F**

aatcagaacaagacttgatgtaaatcgtcgcatttttatctgttttatgttgcaagtatgtttcttgtatattgggtgtttgatattcatataataaaaA

**VCA0094Bx1R**

ttttattatatgaatatcaaacacccaatatacaagaaacatacttgcaacataaaacagataaaaatgcgacgatttacatcaagtcttgttctgattA

**ncRNA59Bx1&2F**

*AGTCACTTCTGAAGCCAACTCTGCATTCTTGCACAGCGCCATAAAATCACCCGCTTGAGCACGAGTGGCACGGTATTCAGGGAGATAACGCCCCGCTTG*A

**ncRNA59Bx1&2F**

CAAGCGGGGCGTTATCTCCCTGAATACCGTGCCACTCGTGCTCAAGCGGGTGATTTTATGGCGCTGTGCAAGAATGCAGAGTTGGCTTCAGAAGTGACTA

**VC1193Bx1F**

tgatcaagctgcgctacaaacagttatcgaaaatttatcaccctgacctgcatggcagcgaagaagagatgaagcgcttaaatagcgcggtaaaaatcgA

**VC1193Bx1R** cgatttttaccgcgctatttaagcgcttcatctcttcttcgctgccatgcaggtcagggtgataaattttcgataactgtttgtagcgcagcttgatcaA

**VC0123Bx1F**

aaaaacagtgcagtgaatttctttttcattttgcatatcgtgcttattgaatcaatgccccctataatcgcaccacgattaggaaaagcaataggatagA

**VC0123Bx1R**

ctatcctattgcttttcctaatcgtggtgcgattatagggggcattgattcaataagcacgatatgcaaaatgaaaaagaaattcactgcactgtttttA

**VC0486BX1F**

tccaaaactgaaagtctttcaacagatttagtcattttatgactgactgtttgatcaaaaatttcagaaaaatcgccagttagcggcagtatggcgcggA

**VC0486BX1R**

CcgcgccatactgccgctaactggcgatttttctgaaatttttgatcaaacagtcagtcataaaatgactaaatctgttgaaagactttcagttttggaA

**VC0486BX2F**

ctttaaTTGTGTTTTATAATGTCGAAACGAAATACACAACTGAGAAGGCACACAATAGTTAAGCTGGTTAATGAACAAGGTGAGGTGAGTGTTGAGGCTA

**VC0486BX2R**

AGCCTCAACACTCACCTCACCTTGTTCATTAACCAGCTTAACTATTGTGTGCCTTCTCAGTTGTGTATTTCGTTTCGACATTATAAAACACAAttaaagA

**VC0916BX2F**

ataaataaaactactttcattttactgctttagttctaattaatatccagctcaattatgagacagacattattggtaagcttgggatactctgataatA

**VC0916BX2R**

attatcagagtatcccaagcttaccaataatgtctgtctcataattgagctggatattaattagaactaaagcagtaaaatgaaagtagttttatttatA

**VCA997BX1F** agctgatgcagggcaaaaccgtgattgcgattgcccaccgtctgtcgaccatcgcagcgatggatcgcctgatcgtgctcgataaaggccaaattgttgA

**VCA997BX1R**

caacaatttggcctttatcgagcacgatcaggcgatccatcgctgcgatggtcgacagacggtgggcaatcgcaatcacggttttgccctgcatcagctA
