# Supplementary material for: Expansion of the SOS regulon of Vibrio cholerae through extensive transcriptome analysis and experimental validation
Source: BMC Genomics. 2018 May 21;19:373. doi: 10.1186/s12864-018-4716-8 (PMC5963079; doi:10.1186/s12864-018-4716-8)
Supplement: Supplementary file 9 — EMSA performed between LexA protein and various promoter region in presence (+) and absence (−) of LexA protein and with a LexA box DNA control (SOS-Box). (PPTX 40364 kb) [file 12864_2018_4716_MOESM9_ESM.pptx]

## Slide 1
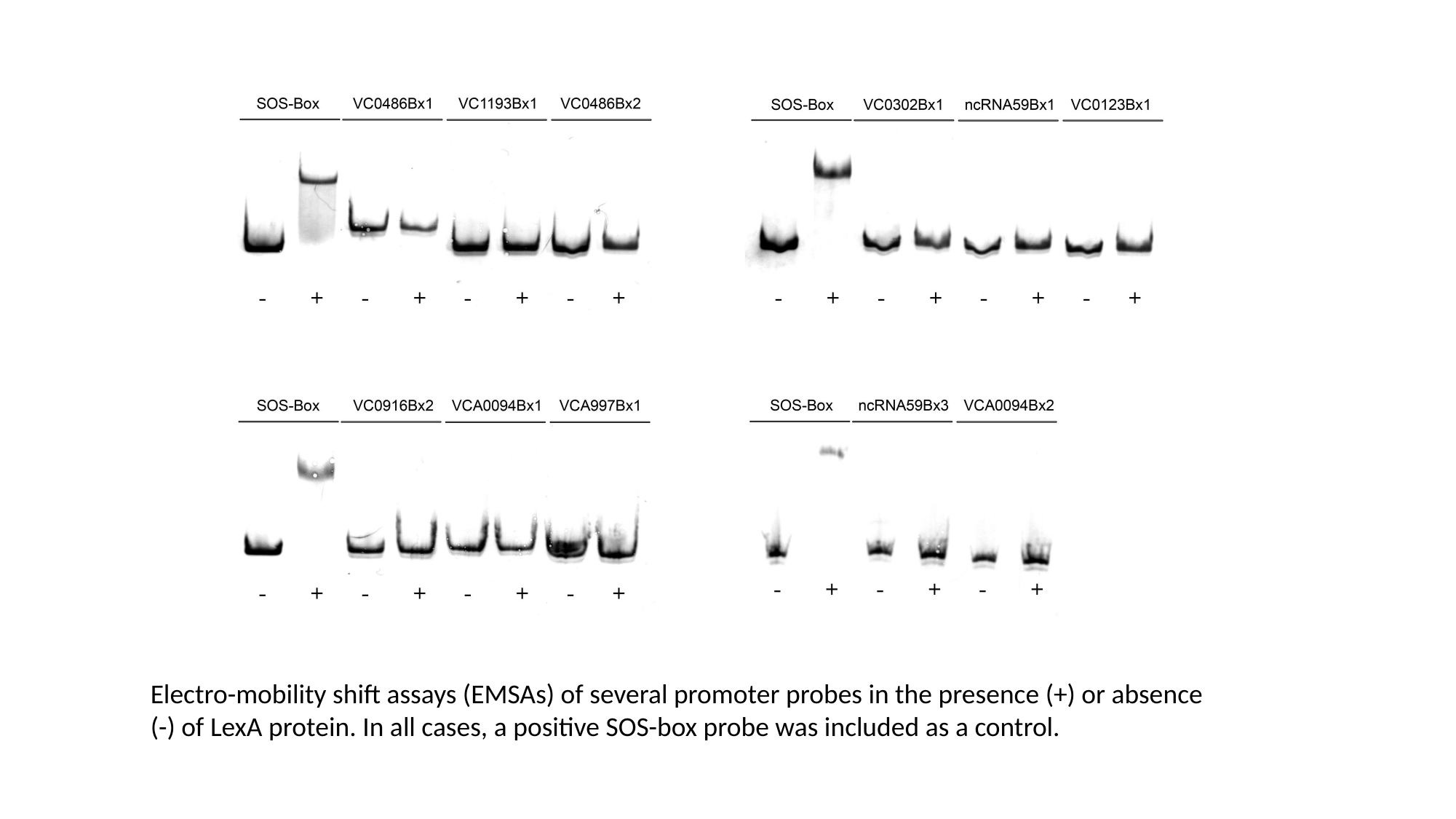

Electro-mobility shift assays (EMSAs) of several promoter probes in the presence (+) or absence (-) of LexA protein. In all cases, a positive SOS-box probe was included as a control.
